#  How I use Transcranial Doppler

**DOI:** 10.1186/s13054-019-2700-6

**Published:** 2019-12-23

**Authors:** Chiara Robba, Fabio Silvio Taccone

**Affiliations:** 1 Policlinico San Martino, IRCCS per l’Oncologia e Neuroscienze, Dipartimento di Scienze Chirurgiche e Diagnostiche Integrate, Università degli Studi di Genova, Genova, Italy; 2 Department of Intensive Care Medicine, Erasme Hospital, Université Libre de Bruxelles (ULB), Route de Lennik, 808, 1070 Brussels, Belgium

**Keywords:** Transcranial Doppler, Flow velocity, Cerebral blood flow, Neurointensive care

## Introduction

**Fig. 1:**
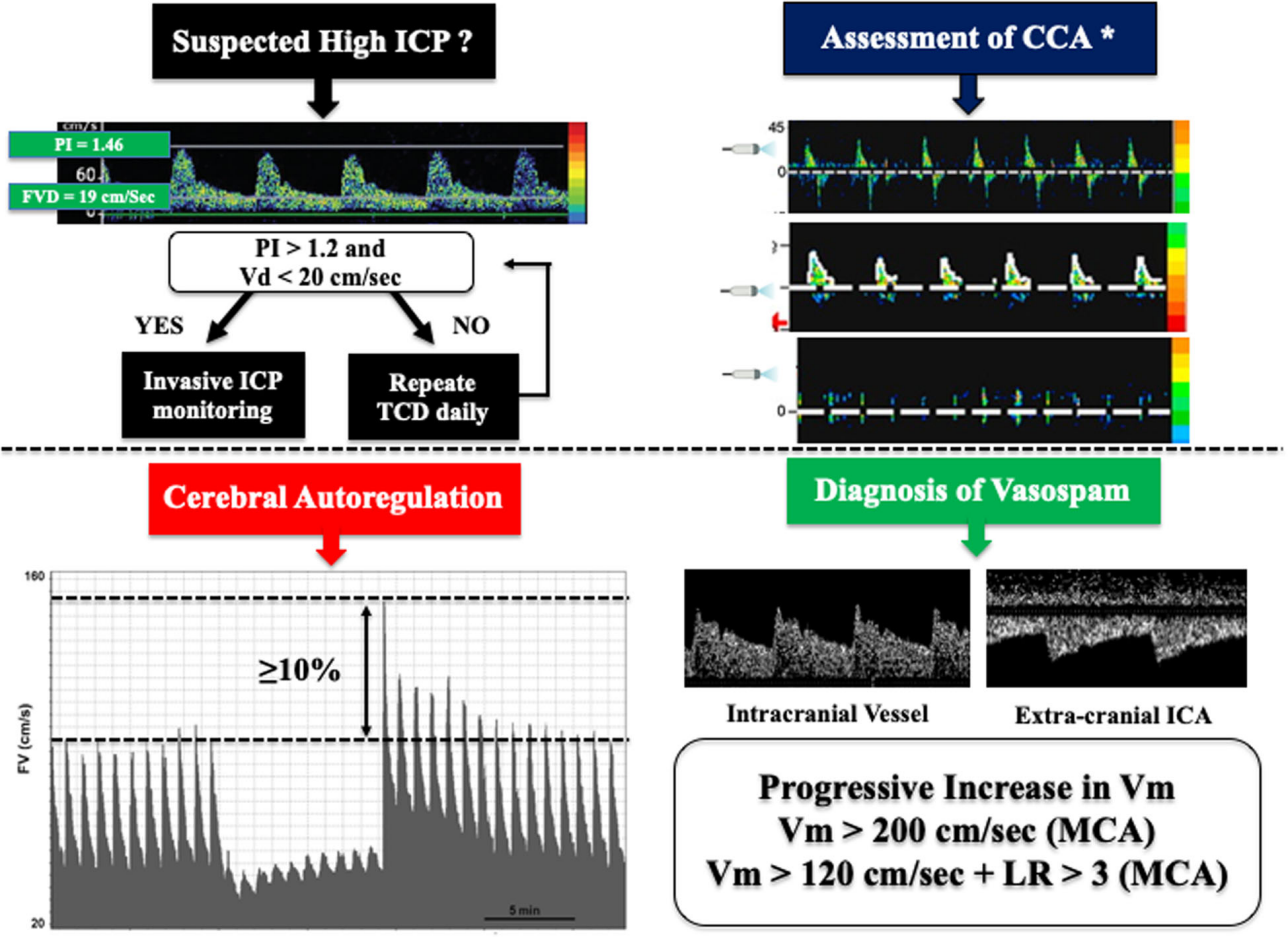
Simplified algorithms on the use of TCD to assess intracranial hypertension, brain death, autoregulation, and cerebral vasospasm in clinical practice. PI, pulsatility index; Vd, diastolic flow velocity; Vm, mean flow velocity; Vs, systolic flow velocity; LR, Lindegaard ratio; CCA, cerebral circulatory arrest. *The three reported images represent reverberating flow (top), systolic pikes (middle), and no flow (bottom), respectively

Transcranial Doppler (TCD) is a bedside, low-cost, and non-invasive technique able to evaluate cerebral hemodynamics [1]; the implementation of transcranial color-coded duplex sonography (TCCS) aids in evaluating the brain anatomy and intracranial lesions [2], real-time monitoring of “basic” (flow velocity (FV) and pulsatility index (PI)) as well as “advanced” TCD-derived parameters (Table 1; Fig. 1). In practice, we use a 2-MHz probe, and most information is obtained by insonating the middle cerebral artery through the temporal window; other windows include the transorbital, occipital, and submandibular windows. TCCD has the advantage to provide a direct visualization of the cerebral anatomy vessels and allow angle correction to assess FV [2]. TCD/TCCD practice is part of the standard training in our institution, and examinations are routinely performed by the medical staff.

**Table 1:** Common parameters derived from transcranial Doppler

	Abbreviation or formula	Normal values	Elevated ICP	Brain death	Cerebral autoregulation	Cerebral vasospasm
Pulsatility index	PI = (sFV − dFV)/mFV	< 1.4	> 1.4	–	–	–
Mean FV	mFV	60–80 cm/s [2]	–	–	–	Mild ≥ 120 cm/s Moderate = 120–200 cm/s Severe ≥ 200 cm/s (with LR < 3)
Diastolic FV	dFV	> 20 cm/s	< 20 cm/s	Negative or absent	–	Increased
Mean flow index	Mx	< 0.3	> 0.3	–	> 0.3 (impaired)	> 0.3
Lindegaard ratio	LR = mFV MCA/mFV extracranial ICA	< 3	–	–	–	Mild ≥ 3 Moderate = 3–6 Severe ≥ 6
THR test					Less than 10% increase from baseline sFV (impaired)	

*FV* flow velocity, *MCA* middle cerebral artery, *ICA* internal carotid artery, *Mx* mean flow index, *dFV* diastolic flow velocity, *mFV* mean flow velocity, *sFV* systolic flow velocity, *CA* cerebral autoregulation, *THR* transient hyperemic test

We discussed herein on how we use TCD in neuro-critically ill patients for hemodynamic indications; some of these proposals could also be used in non-brain injured critically ill patients at a high risk of cerebral complications.

## Non-invasive assessment of intracranial pressure

When the indications for invasive intracranial pressure (ICP) monitoring are met, we recommend intraparenchymal or intraventricular probes, as TCD cannot substitute invasive ICP measurement [3]. However, when indications are unclear or invasive methods are not available (i.e., low-income countries) or contraindicated (i.e., severe coagulopathy), we use TCD as a “triage” tool to non-invasively discriminate patients who are at risk of developing intracranial hypertension [1, 4, 5]. We do not rely on only PI (i.e., PI > 1.4), because other conditions (Additional file 1: Table S1) could affect this parameter [2]. As such, after having considered these conditions, we use the combination of elevated PI and low diastolic FV (< 20 cm/s) to suggest elevated ICP at the bedside. Moreover, we also estimate ICP using formulas combining FV and blood pressure [5, 6], but only as “confirmatory” findings before additional validation of their accuracy will be available. Finally, we perform repeated TCD assessment rather than a single examination (i.e., every 1–2 h) to better understand the changes in the brain hemodynamics following an increase in ICP or after specific ICP-directed therapies.

## Diagnosis of brain death

Although the diagnosis of brain death is based on neurological examination, we use routinely TCD as an ancillary test to demonstrate the absence of cerebral blood flow (CBF) [7]. We use one of the following TCD patterns to determine “cerebral” circulatory arrest (CCA): reverberating flow, systolic spikes, and disappearance of previously recorded FV [2, 7]. According to local practices, when we perform TCD and analyze the waveforms suggesting impending CCA, all the vessels of the circle of Willis through the trans-temporal and occipital windows are examined, as only the detection of the abovementioned flow patterns in all the major intracranial vessels is consistent for brain death [8]. When no intracranial signal is found but brain death criteria are met, we perform a brain CT perfusion or angiography to detect CCA.

## Cerebral autoregulation

We assess cerebral autoregulation (CA) at the bedside as altered CA is related with a poor outcome in many diseases and may increase the risk of cerebral damage [9]. In case of impaired CA, we use TCD to target blood pressure to a level corresponding to the patient’s individual optimal autoregulatory status. The most simple methods to assess CA at the bedside are (a) the static autoregulatory index [9], which is obtained by calculating the percentage of changes in cerebrovascular resistance (CVR = mean arterial pressure/mean FV) after changes in arterial blood pressure, or (b) the transient hyperemic response test (if there are no risks of embolism or hemodynamic instability), which is obtained by compressing the carotid artery and calculating the percentage of change in systolic FV from the baseline (an increase ≥ 10% is considered as intact CA) [10]. Clinicians have to consider that the monitoring of dynamic autoregulation, using the mean flow index (Mx), which is calculated as the correlation coefficient indices between FV and CPP during spontaneous fluctuations in blood pressure, would be more accurate to assess CA [11]. However, this method requires a specific software and a higher competency to interpret the data to improve patients’ management.

## Cerebral vasospasm

Detection of cerebral vasospasm following aneurysmal subarachnoid hemorrhage (SAH) is crucial as this is one of the main determinants of delayed cerebral ischemia and poor neurological outcome in this setting [12]. Although angiography remains the gold standard, we use TCD daily to assess vasospasm, to guide additional investigations, and to monitor the clinical treatment. Indeed, we evaluate the constriction of the cerebral vessels that is associated with a progressive increase of mean FV [13]. In daily practice, we perform serial TCD examinations (one to two/day) in all SAH patients, together with close neurological clinical monitoring; we use TCD for the assessment of all main intracranial vessels and, using TCCD, investigate different segments of such vessels, as vasospasm could be extremely localized. In the presence of clinical suspicious of vasospasm (i.e., neurological deterioration), we use the cutoff of MCA mean flow velocity (mFV) > 200 cm/s [14] to immediately initiate therapy and perform additional confirmatory imaging tests (i.e., cerebral CT perfusion or angiography). If mFV > 120 cm/s and < 200 cm/s, we assess the mFV in the extracranial internal carotid artery using the submandibular window and calculate the Lindegaard ratio (LR; Table 1) to differentiate vasospasm from cerebral hyperemia [15]. As TCD has a sensitivity of 90% (95% confidence intervals [CIs] 77–96%), specificity of 71% (95% CI 51–84%), positive predictive value of 57% (95% CI 38–71%), and negative predictive value of 92% (95% CI 83–96%) to diagnose vasospasm of MCA [13], we still perform cerebral CT perfusion or angiography in case of clinical suspicion of vasospasm with mFV below < 120 cm/s. For other intracranial vessels, in the absence of validated mFV cutoffs, we combine clinical examination, repeated TCD showing a progressive increase in FV, and CT perfusion to detect vasospasm.

## Conclusions

We often use TCD to monitor brain hemodynamics in critically ill patients. Future TCD development, such as the assessment of the compliance of arterial and cerebrospinal fluid compartment as well as critical capillary closing pressure, will further expand its use in this setting [1].

## Supplementary information

**Additional file 1:** **Table S1.** Factors that may influence pulsatility index (PI) and flow velocities. (DOCX 13 kb)

## Data Availability

Not applicable

## Abbreviations

CA: Cerebral autoregulation
CBF: Cerebral blood flow
CPP: Cerebral perfusion pressure
CVR: Cerebrovascular resistance
CT: Computed tomography
FV: Flow velocity
ICP: Intracranial pressure
LR: Lindegaard ratio
mFV: Mean flow velocity
Mx: Mean flow index
NIC: Neurointensive care
PI: Pulsatility index
sARI: Static autoregulatory index
sROR: Static rate of regulation
SAH: Subarachnoid hemorrhage
TCD: Transcranial Doppler
TBI: Traumatic brain injury
