# Supplementary material for: How I use Transcranial Doppler
Source: Crit Care. 2019 Dec 23;23:420. doi: 10.1186/s13054-019-2700-6 (PMC6929281; doi:10.1186/s13054-019-2700-6)
Supplement: Supplementary file 1 — Additional file 1: Table S1. Factors that may influence pulsatility index (PI) and flow velocities. (DOCX 13 kb) [file 13054_2019_2700_MOESM1_ESM.docx]

**Table S1.** Factors that may influence pulsatility index (PI) and flow velocities.

|  | **PI** | **Flow Velocities** |
| --- | --- | --- |
| **Elevate pulse pressure** | Increased |  |
| **Hypercapnia** | Reduced | Increased |
| **Hypocapnia** | Increased | Reduced |
| **Bradycardia** | Increased |  |
| **Elderly population** | Increased |  |
| **Inadequate insonation angle** |  | Reduced |
| **Hypothermia** |  | Reduced |
| **Hyperthermia** |  | Increased |
